# Intraocular pressure-lowering efficacy and safety of bimatoprost 0.03% therapy for primary open-angle glaucoma and ocular hypertension patients in China

**DOI:** 10.1186/1471-2415-14-21

**Published:** 2014-02-25

**Authors:** Kaidi Wang, Li Xu, Zhilan Yuan, Ke Yao, Junmei Zhao, Liang Xu, Aiwu Fang, Mingzhi Zhang, Lingling Wu, Jian Ji, Jiamin Hou, Qing Liu, Xinghuai Sun

**Affiliations:** 1 Eye & ENT Hospital, Shanghai Medical College, Fudan University, Fenyang Road 83, Shanghai, Xuhui District 200031, China; 2 Department of Ophthalmology, the Fourth People’s Hospital of Shenyang, Shenyang, China; 3 Department of Ophthalmology, the People’s Hospital of Jiangsu, Nanjing, China; 4 Eye Center of the 2nd Affiliated Hospital, Medical College of Zhejiang University, Hangzhou, China; 5 Shanxi Eye Hospital, Taiyuan, China; 6 Beijing Institute of Ophthalmology, Beijing Tongren Hospital, Capital University of Medical Science, Beijing, China; 7 Eye Hospital, Wenzhou Medical College, Wenzhou, China; 8 Joint Shantou International Eye Center, Shantou University & the Chinese University of Hong Kong, Shantou, China; 9 Peking University Third Hospital, Peking University Eye Center, Beijing, China; 10 Eye Centre of Tianjin Medical University, Tianjin, China; 11 Eye Institute and Xiamen Eye Center of Xiamen University, Xiamen, China; 12 Allergan Information Consulting (Shanghai) Co., Ltd., Shanghai, China

**Keywords:** Bimatoprost, Glaucoma, Ocular hypertension, Intraocular pressure, Conjunctival hyperemia

## Abstract

**Background:**

To report the clinical outcomes in Chinese patients with primary open-angle glaucoma and ocular hypertension treated with bimatoprost 0.03% therapy.

**Methods:**

Two hundred sixty-three Chinese patients with primary open-angle glaucoma and ocular hypertension who needed initial or additional intraocular pressure (IOP) lowering were recruited in this prospective, open-label, multicenter clinical study and were treated with bimatoprost 0.03%. Patients received bimatoprost 0.03% as initial, replacement or adjunctive IOP-lowering therapy, and follow-up visits were performed at week 1, and month 1 and 3 of the bimatoprost treatment. The efficacy outcome measure was the post-treatment IOP level. The safety outcome measures included the rate of medication-related symptoms, physical signs, reported adverse events, and the level of conjunctival hyperemia.

**Results:**

Among 240 patients who could be categorized by pre-existing therapies and the bimatoprost therapy regimen in the study, IOP values observed in all medication conditions showed significant IOP reduction at all study visits compared with baseline. At 3 months, 8.0 ± 3.7 mmHg (32.0%) reduction in IOP was observed in treatment-naive patients after bimatoprost monotherapy; in the patients previously on various therapy regimens, 1.9 ± 2.8 mmHg (9.5%) to 6.4 ± 6.1 mmHg (24.8%) additional IOP lowering was achieved after switching to bimatoprost monotherapy or bimatoprost combination therapy. The most common adverse event was conjunctival hyperemia, mainly of trace and mild intensity.

**Conclusions:**

Our results show that bimatoprost 0.03% was effective in lowering IOP with favorable safety in Chinese primary open-angle glaucoma and ocular hypertension patients.

## Background

Glaucoma is a major cause of blindness in the world, and the increasing number of cases along with an aging population makes it a huge epidemiologic challenge [1,2]. Conclusive evidence from many well-conducted clinical trials have shown that intraocular pressure (IOP) reduction is beneficial to control the glaucomatous damage for either glaucoma or ocular hypertension patients [3-7].

To date, the prostaglandin/prostamide (PG/PM) class of medications has been shown to be highly effective in IOP lowering with favorable tolerance and hence are used as the first-line ocular antihypertensive agents for glaucoma treatment in many countries [8]. Among these medications, the PM bimatoprost represents a pharmacologically distinct drug that has been approved on the United States market since 2001 for glaucoma and ocular hypertension (OHT) treatment. Bimatoprost lowers IOP by increasing both uveoscleral and trabecular aqueous humor outflow [9]. This hypotensive effect has been consistently proven by numerous clinical studies, and results demonstrated that bimatoprost provides greater or similar reduction in IOP compared with latanoprost and travoprost over the course of 24 hours and over the long term [10-19]. Bimatoprost is generally well tolerated with common adverse effects of mild conjunctival hyperemia, eyelash growth and ocular pruritus [9,20]. In some countries, but not yet in China, a new 0.01% formulation of bimatoprost is available; bimatoprost 0.01% has been demonstrated to have efficacy equivalent to the original bimatoprost 0.03% formulation and be associated with less frequent and less severe conjunctival hyperemia [21].

As the data on clinical outcomes of bimatoprost in Chinese glaucoma and OHT patients are sparse [19,22], this study was designed to evaluate the IOP-lowering efficacy and safety of bimatoprost 0.03% therapy in OHT and primary open-angle glaucoma (POAG) patients in a “real world” clinical setting in China.

## Methods

### Patients

Eligible patients were those who were either previously untreated or treated with POAG or OHT and who required initial or further IOP-lowering therapy in the judgment of the treating physician. The diagnosis of POAG and OHT was made based on the criteria listed in the European Glaucoma Society Guidelines [23]. A baseline IOP of less than 35 mmHg was required for study entry. Patients were only eligible if they were 18 years or older and had not received topical ophthalmic bimatoprost within the 3 months prior to the enrollment.

Key exclusion criteria included uncontrolled systemic disease; known hypersensitivity or contraindications to any components of the study medications; obvious ocular diseases other than POAG or OHT; any corneal lesions precluding accurate tonometry reading; a history of surgery for glaucoma or cataract treatment or any other intraocular surgery within past 3 months; recent use of systemic medications of potential IOP impact; systemic or topical steroid therapy within the previous 21 days or during the study period; participation in other drug or devices trials within 30 days before the enrollment; having pregnancy planned or being pregnant or nursing; and any other condition that, in the investigator’s opinion, might be harmful to the patient or influence the outcomes.

### Study design and treatment

This was a prospective, open-label, multicenter clinical study involving POAG and OHT patients who needed further IOP lowering, in whom bimatoprost was used either as monotherapy or as added medication to the pre-existing therapy regimen. The study was conducted at 11 clinical sites across Northern, North Eastern, Eastern, and Southern China from January 2010 to June 2011. The study protocol was approved by a centralized ethics committee, the Ethics Committee of Eye & ENT Hospital, Shanghai Medical College, Fudan University, Shanghai, China, and this approval was accepted at each site. The study was conducted in compliance with the Declaration of Helsinki and Good Clinical Practices (GCP). All recruited patients provided written informed consent.

Demographic characteristics, medical history, and ophthalmic history were recorded prior to initiation of bimatoprost medication. Ocular examination including visual acuity test, slit-lamp biomicroscopy, tonometry, and conjunctival hyperemia evaluation was performed at baseline and all study visits. IOP was measured with a Goldmann applanation tonometer between 8 am and 10 am, and the mean of 3 readings (5 min apart) was recorded for analyses. The eligible eye was determined as the eye with higher IOP at baseline, or the right eye if both eyes had the same IOP. Conjunctival hyperemia was evaluated with slit-lamp examination, which was performed before tonometry.

Without a wash-out phase, all patients received 1 drop of bimatoprost 0.03% (Lumigan 0.03%, Allergan, Irvine, California, USA) once daily in the evening over the 3-month observation period. Usage of any concurrent non-PG IOP-lowering eye drops remained unchanged. Follow-up data including the ophthalmic examination results, the treatment regimen, and all adverse events were collected at the end of week 1, and months 1 and 3. Patients were withdrawn if they became pregnant or required a change of the treatment, or if the IOP was lower than 6 mmHg.

The study outcome for efficacy was mean IOP level at week 12. Safety outcomes included medication-related symptoms, physical signs, reported adverse events, and the level of conjunctiva hyperemia, which was graded on biomicroscopic examination by the investigator on a 5-point hyperemia grading scale: 0 = none, 0.5 = trace, 1 = mild, 2 = moderate, 3 = severe. The standard color photographs on the Allergan bulbar hyperemia grading guide were used as a comparison scale for the conjunctival hyperemia grading. Photographs of the conjunctiva were taken at the slit lamp as documentation.

### Statistical analysis

All continuous variables are presented as mean ± standard deviation (SD) for the data with normal distribution, and categorical variables are shown as number (%). In the comparison between the mean IOP of each visit and baseline, and the comparison between the mean IOP drop from baseline at the month-1 visit and month-3 visit in each group, paired-sample *t*-test was performed for data of normal distribution, and Wilcoxon’s signed rank test was performed for data of a non-normal distribution. To identify possible risk factors for conjunctival hyperemia of grades 2 and 3, univariate logistic regression analysis was carried out to determine the contribution of possible risk factors including age, sex, diagnosis of POAG or OHT, history of laser treatment for glaucoma, history of surgical treatment for glaucoma, history of comorbidities (i.e., cardiovascular disease and hypertension), blood pressure, and pre-study ocular medications. Multivariate logistic regression models were developed including factors with *P* values of < 0.25. The estimated relative risk of hyperemia in the model was represented by the odds ratio (OR) with a 95% confidence interval (CI). All statistical analyses were performed with SAS 8.0 (SAS Institute Inc., Cary, North Carolina, USA). A *P* value < 0.05 was considered statistically significant.

## Results

### Study population and patient demographics

A total of 263 eligible patients were enrolled in the study for bimatoprost therapy. Overall, the mean age of the study population was 48.7 years, and 58.9% of patients were male. The diagnoses of POAG and OHT presented in 81.4% and 18.6% of the 263 patients, respectively. The most common medical conditions in addition to glaucoma at baseline included history of laser treatment for glaucoma (9.5%), history of surgical treatment for glaucoma (21.8%), and systemic comorbidities (12.9%). Among 263 eligible patients, 240 patients were categorized by pre-existing therapies and the bimatoprost therapy regimen in the study, with Group A: treatment-naive patients who received in-trial bimatoprost monotherapy; Group B: pretrial PG mono-treated patients who received in-trial switch monotherapy with bimatoprost; Group C: pretrial non-PG mono- or combination treated patients who received in-trial switch monotherapy with bimatoprost; Group D: pretrial PG combination-treated patients who received in-trial PM replacement with bimatoprost; Group E: pretrial non-PG mono- or combination treated patients who received bimatoprost as an adjunctive agent in addition to the previous treatment modality (Table 1). There was an undefined group including 19 patients with no follow-up data, 1 newly diagnosed patient who received bimatoprost combination therapy, and 3 patients who altered their therapy over the 3-month study period. All enrolled patients’ characteristics at baseline stratified by treatment condition are also summarized in Table 2.

**Table 1:** Distribution of patients by pre-existing therapies and the bimatoprost therapy regimen in the study, ITT population

**Baseline therapy**	**Bimatoprost therapy in the study**^**a**^	
	**Bimatoprost monotherapy**	**Bimatoprost combination therapy (2–3 types)**
None	89 (33.8%)	NA
	Group A	
PG monotherapy	33 (12.5%)	NA
	Group B	
PG combination therapy	NA	18 (6.8%)
		Group D
Non-PG monotherapy or combination therapy	67 (25.5%)	33 (12.5%)
	Group C	Group E

^a^Data are presented as n (%).

*PG* prostaglandin.

**Table 2:** Patient characteristics at baseline

**Characteristic**^**a**^	**All enrolled N = 263**	**A N = 89**	**B N = 33**	**C N = 67**	**D N = 18**	**E N = 33**	**Undefined N = 23**
Age (years)	48.7 ± 16.6	44.6 ± 15.3	48.2 ± 20.5	52.1 ± 15.7	52.8 ± 12.5	54.1 ± 18.2	44.4 ± 14.7
Sex (male)	155 (58.9%)	47 (52.8%)	19 (57.6%)	45 (67.2%)	6 (33.3%)	22 (66.7%)	16 (69.6%)
Diagnosis							
POAG	214 (81.4%)	53 (59.6%)	31 (93.9%)	64 (95.5%)	18 (100.0%)	32 (97.0%)	16 (69.6%)
OHT	49 (18.6%)	36 (40.4%)	2 (6.1%)	3 (4.5%)	0	1 (3.0%)	7 (30.4%)
Enrolled eye (OD)	141 (53.6%)	44 (49.4%)	17 (51.5%)	36 (53.7%)	13 (72.2%)	17 (51.5%)	14 (60.9%)
History of laser treatment for glaucoma	25 (9.5%)	7 (7.9%)	6 (18.2%)	6 (9.0%)	3 (16.7%)	1 (3.0%)	2 (8.7%)
History of surgical treatment for glaucoma	57 (21.8%)	10 (11.5%)	7 (21.2%)	14 (20.9%)	8 (44.4%)	11 (33.3%)	7 (30.4%)
Comorbidities	34 (12.9%)	4 (4.5%)	11 (33.3%)	10 (14.9%)	2 (11.1%)	7 (21.2%)	0

^a^Continuous data are presented as mean ± SD and categorical data are presented as n (%).

*POAG* primary open-angle glaucoma; *OHT* ocular hypertension. Group A: treatment-naive patients received in-trial bimatoprost monotherapy; Group B: pretrial PG mono-treated patients received in-trial switch monotherapy with bimatoprost; Group C: pretrial non-PG mono- or combination-treated patients received in-trial switch monotherapy with bimatoprost; Group D: pretrial PG combination-treated patients received in-trial PM replacement with bimatoprost; Group E: pretrial non-PG mono- or combination-treated patients received bimatoprost as an adjunctive agent in addition to the previous treatment modality.

Two hundred and three patients (77.2%) completed all study visits as planned, and 60 patients dropped out due to losses to follow-up (34/263, 12.9%), protocol violation (14/263, 5.3%), and adverse events (13/263, 4.9%).

### IOP-lowering effects

IOP in the 23 patients in the undefined group was not analyzed due to invalid data. For the other groups (Groups A to E), all IOP data presented for the bimatoprost therapy efficacy evaluation are from intent-to-treat (ITT) analyses.

Mean baseline IOP values of the patients were 24.6 ± 4.4 mmHg in Group A, 18.6 ± 3.4 mmHg in Group B, 19.8 ± 4.0 mmHg in Group C, 21.4 ± 5.3 mmHg in Group D, and 23.6 ± 5.7 mmHg in Group E, respectively.

**Figure 1:**
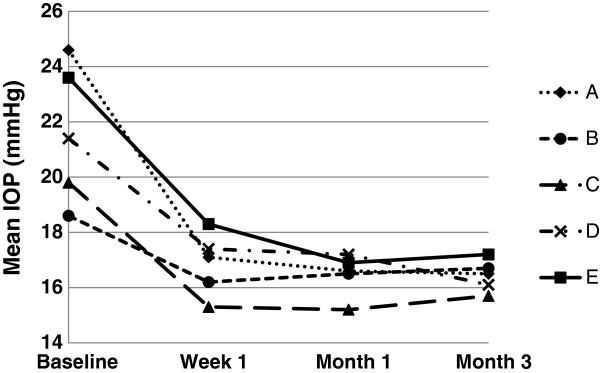
**Mean IOP of the patients in groups A through E, ITT population.** Group A: treatment-naive patients received in-trial bimatoprost monotherapy; Group B: pretrial PG mono-treated patients received in-trial switch monotherapy with bimatoprost; Group C: pretrial non-PG mono- or combination-treated patients received in-trial switch monotherapy with bimatoprost; Group D: pretrial PG combination-treated patients received in-trial PM replacement with bimatoprost; Group E: pretrial non-PG mono- or combination-treated patients received bimatoprost as an adjunctive agent in addition to the previous treatment modality.

Following bimatoprost treatment, IOP values observed in all groups were reduced as early as 1 week and were maintained during the study period of 3 months (Figure 1). Significant reductions in mean IOP were observed in all treatment groups at week 1, month 1, and month 3 when compared to baseline (*P* < 0.05), with mean IOP ranging from 15.7 ± 2.6 mmHg (Group C) to 17.2 ± 4.4 mmHg (Group D) at month 3.

Moreover, the mean IOP changes from baseline of all groups were significant (*P* < 0.05). In comparisons of the level of IOP reduction between visits, data from week 1 were not included since there was no washout phase before the bimatoprost treatment. Therefore, only a comparison between the second and third follow-up visits was performed, and results showed no significant difference in the groups except for Group D, indicating a consistent IOP-lowering effect of bimatoprost during the study period. The greatest mean IOP change was found in Group A, with a highly significant mean IOP change of -7.4 ± 3.8 mmHg (-29.7%) at week 1, -8.0 ± 3.3 mmHg (-32.0%) at month 1, and -8.0 ± 3.7 mmHg (-32.0%) at month 3 (all *P* < 0.0001 compared with baseline). The smallest mean IOP change presented in Group B, with a mean IOP change of -2.4 ± 2.5 mmHg (-12.3%) at week 1, -2.1 ± 2.9 mmHg (-10.7%) at month 1, and -1.9 ± 2.8 mmHg (-9.5%) at month 3 (all *P* < 0.001 compared with baseline) (Table 3).

**Table 3:** Mean IOP change from baseline and mean percentage change for groups A to E at each visit

**IOP parameter**^**a**^		**A N = 89**	**B N = 33**	**C N = 67**	**D N = 18**	**E N = 33**
Baseline	Mean IOP, mmHg	24.6 ± 4.4	18.6 ± 3.4	19.8 ± 4.0	21.4 ± 5.3	23.6 ± 5.7
1 week	IOP change, mmHg	-7.4 ± 3.8***	-2.4 ± 2.5***	-4.5 ± 3.6***	-4.0 ± 4.8**	-5.2 ± 4.8***
	Percentage change	-29.7 ± 13.7	-12.3 ± 11.9	-21.7 ± 16.0	-15.6 ± 18.6	-20.8 ± 18.0
1 month	IOP change, mmHg	-8.0 ± 3.3***	-2.1 ± 2.9***	-4.6 ± 3.6***	-4.2 ± 5.2*	-6.7 ± 6.1***
	Percentage change	-32.0 ± 10.2	-10.7 ± 15.5	-21.4 ± 15.5	-16.2 ± 20.8	-25.5 ± 22.7
3 months	IOP change, mmHg	-8.0 ± 3.7***	-1.9 ± 2.8***	-4.1 ± 3.5***	-5.3 ± 5.1***	-6.4 ± 6.1***
	Percentage change	-32.0 ± 11.8	-9.5 ± 13.2	-18.9 ± 16.1	-21.7 ± 19.5	-24.8 ± 20.2
*P* value^b^		0.6975	0.4192	0.1574	0.0279	0.6219

*P < 0.05, **P < 0.01, ***P < 0.001 compared with baseline IOP in this group using paired-sample *t*-test or Wilcoxon’s signed rank test.

^a^Data are presented as mean ± SD.

^b^Data comparison between the month-1 visit and month-3 visit in each group using paired-sample *t*-test or Wilcoxon’s signed rank test.

*IOP* intraocular pressure.

Group A: treatment-naive patients received in-trial bimatoprost monotherapy; Group B: pretrial PG mono-treated patients received in-trial switch monotherapy with bimatoprost; Group C: pretrial non-PG mono- or combination-treated patients received in-trial switch monotherapy with bimatoprost; Group D: pretrial PG combination-treated patients received in-trial PM replacement with bimatoprost; Group E: pretrial non-PG mono- or combination-treated patients received bimatoprost as an adjunctive agent in addition to the previous treatment modality.

### Adverse events

Thirteen out of 263 enrolled patients failed to complete at least one follow-up visit due to administrative reasons or loss to follow-up. All other 250 patients were included in analyses of adverse events. Generally, the study regimens with bimatoprost were well tolerated and no serious adverse events were reported. The incidence and types of treatment-related adverse events at each visit are listed in Table 4.

**Table 4:** Summary of adverse events excluding conjunctival hyperemia

**AE**	**Number (%)**			
	**Week 1 N = 250**	**Month 1 N = 232**	**Month 3 N = 212**	**Over the study N = 250**
Eyelash growth	4 (1.6%)	16 (6.9%)	17 (8.0%)	23 (9.2%)
Skin pigmentation	5 (2.0%)	11 (4.7%)	12 (5.7%)	16 (6.4%)
Ocular pruritus	5 (2.0%)	9 (3.9%)	3 (1.4%)	12 (4.8%)
Foreign body sensation	6 (2.4%)	6 (2.6%)	1 (0.5%)	11 (4.4%)
Dry eyes	5 (2.0%)	6 (2.6%)	1 (0.5%)	9 (3.6%)
Conjunctivitis	1 (0.4%)	2 (0.9%)	1 (0.5%)	3 (1.2%)
Eye swelling pain	3 (1.2%)		1 (0.5%)	4 (1.6%)
Lachrymation	2 (0.8%)			2 (0.8%)
Ocular burning sensation	1 (0.4%)	1 (0.4%)		2 (0.8%)
Eye sore	1 (0.4%)			1 (0.4%)
Ocular stinging	2 (0.8%)	1 (0.4%)		3 (1.2%)
Ocular abnormal sensation		1 (0.4%)		1 (0.4%)
Ocular discharge	1 (0.4%)	1 (0.4%)		2 (0.8%)
Heavy feeling in eyelids		1 (0.4%)		1 (0.4%)
Corneal epithelial ulcer		1 (0.4%)		1 (0.4%)
Trichiasis			1 (0.5%)	1 (0.4%)
Conjunctival follicle		1 (0.4%)		1 (0.4%)
Subconjunctival hemorrhage	1 (0.4%)			1 (0.4%)
Slight visual acuity reduction	1 (0.4%)			1 (0.4%)
Fatigue	1 (0.4%)			1 (0.4%)
Headache	2 (0.8%)		1 (0.5%)	2 (0.8%)
Whole-body pruritus			1 (0.5%)	1 (0.4%)
Overall	28 (11.2%)	38 (16.4%)	32 (15.1%)	59 (23.6%)

*AE* adverse event.

Throughout the study, other than conjunctival hyperemia, the most commonly reported adverse events were eyelash growth, skin pigmentation, ocular pruritus, foreign body sensation, and dry eyes, which occurred in 9.2%, 6.4%, 4.8%, 4.4%, and 3.6% of the 250 patients respectively. All other adverse events occurred in less than 2% of the patients, and most of them disappeared after continuing therapy.

### Conjunctival hyperemia

**Figure 2:**
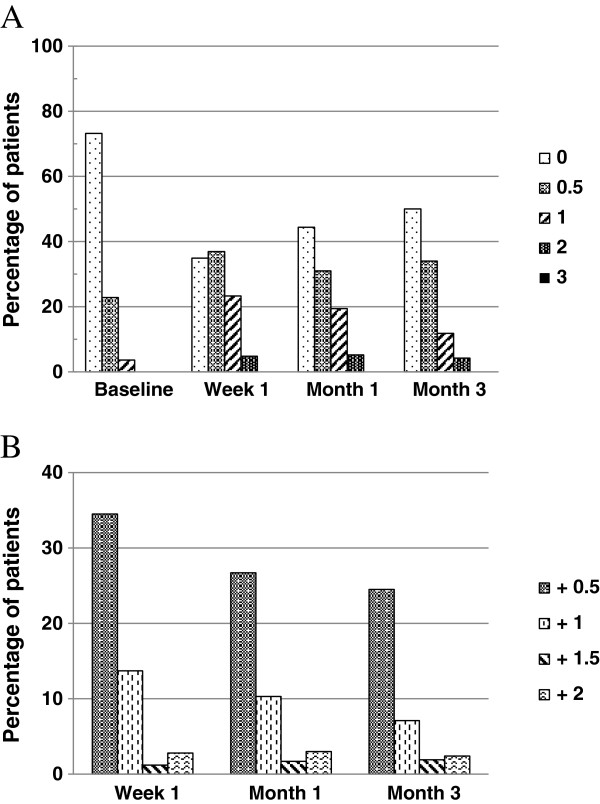
**Conjunctival hyperemia scoring distribution at each visit. A**, Percentage of patients with each grade; **B**, Percentage of patients with conjunctival hyperemia grade increase from baseline n = 250.

Conjunctival hyperemia was the most frequently reported side effect in the bimatoprost-treated patients. However, moderate to severe hyperemia was uncommon. The mean percentage of patients with conjunctival hyperemia Grade 2 (moderate) was 4.8%, 5.2%, and 4.2% of the 250 patients at week 1, month 1, and month 3 respectively; Grade 3 (severe) conjunctival hyperemia was not noted in any patient during the study (Figure 2A). Furthermore, the severity changes of conjunctival hyperemia from baseline at each visit were mainly 0.5-grade increases (Figure 2B).

The analyses of the associations between patient characteristics and moderate to severe conjunctival hyperemia (Grade 2 and 3) are shown in Table 5. In univariate logistic regression models, no parameter was found to be significantly associated with moderate to severe conjunctival hyperemia, although the correlation between sex (higher percentage of men) and moderate to severe conjunctival hyperemia approached significance (*P* = 0.0711). A multivariate regression model using sex and history of laser treatment as factors also identified no significant risk factors for moderate to severe conjunctival hyperemia (data not shown).

**Table 5:** Association between patient characteristics and moderate to severe conjunctival hyperemia (grade 2 and 3) in univariate logistic regression models

	**OR**	**95% CI**	***P*-value**
Age, years	1.338	0.627- 2.856	0.4521
(≥ 50 vs < 50)			
Diagnosis	1.624	0.541- 4.877	0.3871
(POAG vs OHT)			
Sex	2.178	0.935-5.072	0.0711
(male vs female)			
History of laser treatment for glaucoma	0.289	0.038-2.214	0.2321
(yes vs no)			
History of surgical treatment for glaucoma	0.882	0.342-2.275	0.7956
(yes vs no)			
Comorbidities	1.346	0.479-3.783	0.5725
(yes vs no)			
Hypertension	0.663	0.293-1.500	0.3237
(yes vs no)			
Duration of pre-study antiglaucoma medication use	1.257	0.592-2.668	0.5516
(< 1 month vs ≥ 1 month )			

*OR* odds ratio; *CI* confidential interval.

## Discussion

In this multicenter clinical study, bimatoprost 0.3% therapy was shown to provide substantial IOP lowering when used in Chinese patients with glaucoma or OHT. The mean IOP of patients who received either no previous treatment (Group A) or other prior medications (Group B-E) was significantly reduced after just 1 week of bimatoprost treatment and maintained over the 3 months study period. The occurrence of treatment-related adverse events including conjunctival hyperemia was acceptable, with only 13 (4.9%) patients discontinuing the study medication because of adverse events.

Elevated IOP is the most significant risk factor for optic nerve damage and visual field progression in glaucoma patients [24,25]. The Early Manifest Glaucoma Trial (EMGT) and Canadian Glaucoma Study have clearly demonstrated that each 1 mmHg reduction in IOP corresponds to 10% or 19% decrease in the risk of the progression of glaucomatous damage [26,27], suggesting the importance of absolute IOP reduction during glaucoma or OHT management. In the present study, the mean IOP reductions from baseline IOP 3 months after in-trial bimatoprost monotherapy were 8.0 ± 3.7 mmHg (32.0 ± 11.8%) in the treatment-naive patients, 1.9 ± 2.8 mmHg (9.5 ± 13.2%) in pretrial PG mono-treated patients, and 4.1 ± 3.5 mmHg (18.9 ± 16.1%) in pretrial non-PG mono- or combination-treated patients. After in-trial bimatoprost combination therapy, mean IOP reductions at 3 months were 5.3 ± 5.1 mmHg (21.7 ± 19.5%) in pretrial PG combination-treated patients and 6.4 ± 6.1 mmHg (24.8 ± 20.2%) in pretrial non-PG mono- or combination treated patients.

The IOP-lowering effects revealed in our study were similar to the findings of other observational or comparative clinical evaluations. Faridi and associates found a 9.45 mmHg (36%) IOP reduction at 2 months and a 9.23 mmHg (35%) IOP reduction at 6 months after bimatoprost 0.03% monotherapy in newly diagnosed OHT and POAG patients [18]. Quinones and Earl reported that bimatoprost 0.03% monotherapy for glaucoma and OHT provides 4.52 mmHg (21.52%) and 4.22 mmHg (19.63%) additional IOP reduction after switching from topical β-blocker monotherapy at week 6 and week 12, respectively [28]. Moreover, Feuerhake *et al*’s study with the fixed combination of bimatoprost 0.03% and timolol 0.5% for POAG or OHT showed 25.8% and 30.3% IOP lowering after 3 months of replacement of previous β-blocker monotherapy and α2-agonist monotherapy, respectively [29].

Of note, in the group of patients who underwent PG medication treatment prior to the current study, bimatoprost still had a significant effect on IOP lowering from baseline, which was maintained throughout the study. A further 1.9 mmHg (9.5%) and 5.3 mmHg (21.7%) IOP drop was achieved after changing from PG to bimatoprost in the monotherapy and combination therapy regimen groups, respectively, at month 3. Similarly, Kammer and colleagues found an additional 1.9 mmHg and 2.1 mmHg mean diurnal IOP reduction from latanoprost mono-treated baseline in glaucoma or OHT patients after 1 and 3 months of bimatoprost 0.03% monotherapy replacement, respectively, and this IOP-lowering efficacy was significantly stronger when compared with switching to travoprost monotherapy [14]. Data from a 24-month study of OAG patients also demonstrated a 15.0–24.0% additional reduction in IOP after changing latanoprost to bimatoprost as monotherapy or in multi-therapy, before adding a further adjunctive agent [30]. Together these results suggest that bimatoprost may provide better IOP control than PGs, which may be attributed in part to its ocular distribution to outflow tissues and dual outflow mechanism following topical administration [9,31]. Considering its highly cost-effective profile [32,33], bimatoprost represents a promising hypotensive lipid to control the elevated IOP in patients with POAG or OHT, especially when a substitution therapy is needed.

In this study, bimatoprost appeared to be clinically safe and well tolerated since the overall treatment withdrawals rate was low. The type and incidence of the treatment-related adverse events were in agreement with previous studies [34-37], with conjunctival hyperemia the most commonly reported ocular adverse event. It should be pointed out that, despite the relatively high overall incidence, the majority of reported hyperemia cases were trace or mild, and most of the patients with a hyperemia increase from baseline had only a 0.5-grade increment. In addition, no significant associations were found between having moderate to severe conjunctival hyperemia and patient characteristics including age, sex, diagnosis of POAG or OHT, history of laser treatment for glaucoma, history of surgical treatment for glaucoma, comorbidities history, blood pressure, and duration of pre-study antiglaucoma medication use.

The mean age of patients in this study was 49 years. In a previously published large, open-label study of real-world use of travoprost in patients diagnosed with open-angle glaucoma or ocular hypertension in China, the mean age of patients was also 49 years [38]. The reasons that the patients in these studies have tended to be younger than the general glaucoma population are unknown, but beta-blockers continue to be widely used in China because of their good efficacy and low cost. For young patients with a longer remaining life expectancy, physicians may be more likely to recommend a PG or PM because of their superior efficacy, and younger patients may be more willing to accept the higher cost of these medications.

The study completion rate was only 77.2%, primarily because 34 (12.9%) patients were lost to follow-up, including 19 (7.2%) patients who had no post-baseline data and were not included in the efficacy analysis. The patients lost to follow-up were not contacted to determine why they discontinued from the study, and it is possible that some of these patients failed to return for study visits because of adverse effects, lack of efficacy, or dissatisfaction with the study treatment. However, we believe that a more likely explanation for the loss to follow-up is that many patients who traveled to large cities to visit eye centers in hospitals for diagnosis and treatment guidance were unwilling to travel for follow-up. Patients who experienced an intolerable adverse event may have been more likely to return to the eye centers for guidance or a change in the treatment regimen. Another study limitation was that some of the reported adverse effects in patients using more than one IOP-lowering medication may have been caused by the other medications, rather than bimatoprost, though the design of the study makes it difficult to estimate how often this occurred.

Since previous clinical evaluations suggest that glaucoma or OHT patients are rarely troubled by temporary ocular side effects, specifically ocular redness [39,40], the mild hyperemia after bimatoprost treatment did not represent a clinical safety concern. The anticipated introduction in China of the new bimatoprost 0.01% formulation with the same efficacy and improved tolerability as the original bimatoprost 0.03% formulation, as well as education of patients explaining the importance of IOP lowering and drug efficacy, may further improve their acceptance and compliance [41].

## Conclusions

The results of this 3-month multicenter clinical study demonstrate clinically important and statistically significant IOP-lowering efficacy of bimatoprost 0.03% for POAG and OHT patients who were newly diagnosed or requiring medication replacement or additional intervention. Considering the efficacy together with the drug’s favorable tolerability profile, we conclude that the choice of bimatoprost can be considered as first-line treatment for POAG or OHT patients, and as a favorable switch or adjunctive agent for those with a poor response to other ocular hypotensive therapy regimens.

## Competing interests

Kaidi Wang, Li Xu, Zhilan Yuan, Ke Yao, Junmei Zhao, Liang Xu, Aiwu Fang, Mingzhi Zhang, Lingling Wu, Jian Ji, Jiamin Hou, and Xinghuai Sun have no competing interests in bimatoprost or Allergan Information Consulting (Shanghai) Co., Ltd. Qing Liu is employed by Allergan Information Consulting (Shanghai) Co., Ltd.

## Authors’ contributions

KW, LX, ZY, KY, JZ, LX, AF, MZ, LW, JJ, JH, XS: principal investigators for the study involved in study design, data collection, data interpretation, and drafting the manuscript; QL: data interpretation, drafting the manuscript, and critical revision of the manuscript for English and important intellectual content. All authors read and approved the final manuscript.

## Pre-publication history

The pre-publication history for this paper can be accessed here:

http://www.biomedcentral.com/1471-2415/14/21/prepub
